# Production and Characterization of an Integrated Multi-Layer 3D Printed PLGA/GelMA Scaffold Aimed for Bile Duct Restoration and Detection

**DOI:** 10.3389/fbioe.2020.00971

**Published:** 2020-08-26

**Authors:** Yang Xiang, Weijia Wang, Yuanhui Gao, Jianquan Zhang, Jing Zhang, Zhiming Bai, Shufang Zhang, Yijun Yang

**Affiliations:** ^1^Department of Hepatobiliary Surgery, Affiliated Haikou Hospital of Xiangya Medical College, Central South University, Haikou, China; ^2^Department of Urology Surgery, Affiliated Haikou Hospital of Xiangya Medical College, Central South University, Haikou, China; ^3^School of Metallurgy and Environment, Central South University, Changsha, China; ^4^Central Laboratory, Affiliated Haikou Hospital of Xiangya Medical College, Central South University, Haikou, China; ^5^Department of Obstetrics and Gynecology, Affiliated Haikou Hospital of Xiangya Medical College, Central South University, Haikou, China

**Keywords:** 3D printing, IKVAV, hydrogel, bile duct, magnetic resonance imaging (MRI)

## Abstract

We successfully fabricated artificial bile duct via 3D printing technique which was composed of poly (lactic-co-glycolic acid) (PLGA) and gelatin methacrylate (GelMA). The PLGA-inner layer provided sufficient strength to support the bile duct contraction, the GelMA-outer layer possessed good biocompatibility to provide a good living environment for the cells. Moreover, IKVAV laminin peptide (Ile-Lys-Val-Ala-Val) and ultrasmall superparamagnetic iron oxide (USPIO) were used to regulate scaffold cell adhesion and magnetic resonance imaging (MRI) detection, respectively. After BMSCs co-culture with IKVAV at a certain concentration, the survival rate and adhesion of BMSCs was increased obviously. Meanwhile, the fabricated scaffold exhibited the tensile modulus in the range of 17.19 – 29.05 MPa and the compressive modulus in the range of 0.042 – 0.066 MPa, which could meet the needs of human implantation. In an animal experiment *in vivo* pig bile duct regeneration, PLGA/GelMA/IKVAV/USPIO duct conduits could promote bile duct regeneration and enhance cytokeratin 19 (CK19) expression. In summary, the composite bile duct scaffold with excellent MRI imaging function and biocompatibility could be used to develop bioactive artificial bile ducts.

## Introduction

In the current treatment of extrahepatic bile ducts affected by tumors or stenosis, surgical resection and reconstruction often cause postoperative complications, such as biliary leakage, bile duct strictures and bile leakage (Zografakis et al., 2003; Vieira-Silva et al., 2019). Clinical treatment, such as intrabiliary stent placement, percutaneous transhepatic biliary drainage (PTBD) and biliary anastomosis, has been used to relieve obstruction of the common bile duct and repair damaged bile ducts. However, different types of complications using metal and plastic stents (polyethylene, polyvinyl chloride, and polyurethane) have been reported, particularly in association with biofilm accumulation, clogging, and bacterial infection (Bege et al., 2012; Ogura et al., 2019). Therefore, if an artificial bile duct could be obtained that is completely consistent with the function of natural organs, artificial bile duct could be implanted to replace the pathological bile duct to prevent bile flow disorder and avoid the occurrence of liver transplantation. During the process of excellent bile duct preparation, two major factors should be considered.

Material selection is a key factor for bile duct restoration. Among them, poly (L-lactide-co-glycolide) (PLGA) and polycaprolactone (PCL) was a biocompatible (Reid et al., 2013), degradable (Lee et al., 2012), non-toxic material (Li et al., 2015). It has many successful applications in bile duct regeneration (Zong et al., 2017). PLGA could provide sufficient support strength to prevent bile duct contraction and narrowness. Moreover, it has good mechanical properties. In addition, proper flexibility and rigidity could facilitate cell migration. Recently, an emerging photo-cross linkable gelatin methacrylate (GelMA) have recently attracted increasing research interest in tissue engineering (Mccoul et al., 2017; O’Bryan et al., 2017; Grosskopf et al., 2018; Gao et al., 2019), which was not only owing to their biocompatibility of gelatin, but also because of easy cross-linking under UV light irradiation.

The second factor is surface modification and scaffold fabrication (Li et al., 2019). Although numerous materials have been exploited for artificial bile ducts, such as gelatin (Yan et al., 2018), poly (L-lactide-co-glycolide) (PLGA) (Zong et al., 2017), and polycaprolactone (PCL) (Bloise et al., 2020), few studies reported bilayered scaffold for bile duct tissue engineering. Li et al. (2020) reported a novel 3D printing PCL/GelMA scaffold containing USPIO for MRI, which could achieve MRI imaging and be beneficial to the proliferation of cells on the scaffolds, but there was poor cell adhesion properties. As a key factor in bile duct tissue engineering, an ideal scaffold would support cell adhesion, proliferation and differentiation, and act as physical reservoir to support bile flow and load bioactive substances. However, a scaffold composed of one material makes it difficult to strike a balance between the requirements to support bile flow and new tissue formation. Therefore, PLGA tube modification is critical for optimal biocompatibility. Nucleic acids (Kang et al., 2015), polypeptides (Han et al., 2010), and cells (Lee et al., 2015) were commonly used for surface coatings. IKVAV laminin peptide have good hydrophilicity and good biochemical properties, can improve cell adhesion, and is a substrate for bioactive scaffold materials. Therefore it was widely used in the field of tissue engineering. Laminin was found as an extracellular matrix (ECM) molecule in basement membranes, was the key substance for cell adhesion and polarization, widely used in neuroengineering (Silva et al., 2014). Prior study reported that laminin could promote bile duct cell polarization to form bile ducts (Tanimizu et al., 2007). However, there were no studies on the interaction between IKVAV and bile duct cells.

3D bioprinting technology hold great promise in the field of tissue engineering and regenerative medicine (Patel et al., 2017; Roh et al., 2017). This technology has been widely utilized to prepared arbitrary-shape three-dimensional tissues and organs including bile duct-like structures. Herein, layer-by-layer freeform production was used to encapsulate cells within microstructure of biocompatible hydrogel mixtures. Narayanan et al. reported that hydrogels are composed of human adipose-derived stem cells, PLA fibers and sodium alginate to print into a meniscus shape through extrusion bioprinting (Narayanan et al., 2016). Lozano et al. (2015) used a coaxial nozzle extrusion device to print neuron cells into a brain-like structure, and the results showed that the printed nerve cells can stretch and grow synapses. However, the degradation and repair of the 3D printing stent *in vivo* could not be detected in real time. Diagnostic tools, including computed tomography (CT) and magnetic resonance imaging (MRI), were employed to combination with the 3D scaffold to achieve a dual effect of diagnosis and treatment (Wang et al., 2017).

**SCHEME 1:**
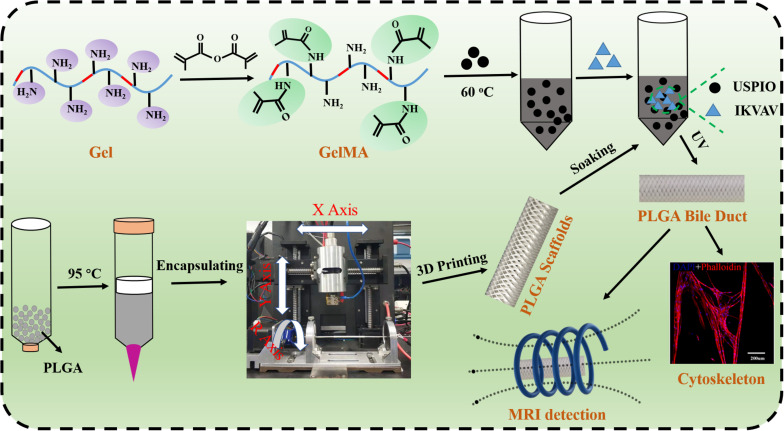
Schematic of GelMA and the schematic illustration of PCL bile duct with LBLC method.

In this study, we innovatively designed a novel bilayered bile duct scaffold by layer by layer casting (LBLC) method as Scheme 1. The inner layer consist of PLGA, which exhibited appropriate mechanical properties, slow degradation kinetics and good biocompatibility. GelMA as matrix to incorporate IKVAV and ultrasmall superparamagnetic iron oxide (USPIO) was used for the outer, which would be conducive to formation of new tissue due to its rapid degradation. To further demonstrate the application, we tested the bioactivity and adhesion of IKVAV and examined the effects of the IKVAV on BMSCs cells. Meanwhile, we evaluated the ability of scaffold for bile duct regeneration *in vivo* pig bile duct defect model. Our engineered scaffold could be used in the construction of an artificial bile duct, due to its high flexibility, suitable mechanical strength and biocompatibility.

## Materials and Methods

### Reagents and Materials

Gelatin (Gel, derived from pig skin, adhesive strength ∼300 g bloom) and methacrylate anhydride (MA) were obtained from Aladdin Reagent Company (Shanghai, China). The initiator lithium phenyl-2,4,6-trimethylbenzoylphosphinate (LAP) was purchased from Yinchang New Material Co., Ltd. (Shanghai, China). IKVAV peptides (C_16_H_31_O-NH-AAAGGGGEIKVAV-COOH, purity at >98%) was purchased from Apeptide Bio-Technology Co., Ltd. (Shanghai, China). Poly (lactic-co-glycolic acid) (PLGA, lactide: glycolide = 75:25, *M*_*w*_ = 66 ∼ 107 kDa) was purchased from shanghai Ding Biological Technology Co., Ltd. (Shanghai, China). USPIO (average particle size = 20 nm) were obtained from Sino Biomaterials Co., Ltd. (Shanghai, China). The other reagents were listed as follows: Dulbecco’s modified Eagle’s medium (DMEM)/F12 with Glutamax (Invitrogen, Carlsbad, CA, United States) and fetal bovine serum (FBS, Gibco, United States), penicillin/streptomycin solution (Gibco, United States), Cell Counting Kit-8 (CCK-8, Dojindo, Japan). All other reagents were analytical reagent grade.

### Synthesis of GelMA

Gelatin (10 g) was dissolved in 100 mL distilled water. After being dissolved at 50°C, 15 mL of methacrylate anhydride was added and reacted at 50°C for 4 h. Then dialysis with dialysis bags (molecular weight cutoff: 1000) for 2–3 days and then lyophilized at 80°C to obtain methacrylate gelatin (GelMA).

### Fabrication of the PLGA Bile Duct Scaffold

All printings were carried out using a customized 3D printer (EFL-BP-6800, Suzhou, China) equipped with a nozzle temperature controller and a chamber temperature controller (Park et al., 2017). Firstly, PLGA particles were put into the barrel of a high-pressure near-field direct writing 3D printer, then set the extrusion temperature to 95°C and the bed temperature to 70°C for 2 h. After the PLGA was melted, air pressure was set to 7 kPa while applying voltage at 4 kV. Finally, the PLGA bile duct model was printed on a metal rotating shaft of 5 mm diameter by using default speed of 1000 mm/min, and the total number of layers was set to 100.

### Fabrication of the PLGA/GelMA/IKVAV/USPIO Composite Bile Duct Scaffold

5 wt% GelMA and 0.5 wt% LAP was dissolved in PBS at 37°C. The IKVAV peptide (200 μg) was then added to the GelMA solution. Finally, GelMA/IKVAV solution and USPIO were mixed in accordance with a different mass/volume (g/L) ratio and fully mixed using a vortex mixer to prepare the final GelMA/IKVAV/USPIO scaffold for subsequent use. The PLGA tubular stent was fully infiltrated with the GelMA/IKVAV/USPIO solution, and then the GelMA/IKVAV/USPIO solution was crosslinked by a 405 nm ultraviolet (UV) lamp with a light intensity of 3.0 mW/cm^2^ for 50 s, and finally the PLGA/GelMA/IKVAV/USPIO solution composite tubular stent was prepared.

### Preparation of Cell-Scaffold Constructs

The bone marrow mesenchymal stem cell (BMSCs) were cultured in stem cell medium supplemented with DMEM/F12 with GlutaMAX and 10% fetal bovine serum, and were cultured under 5% carbon dioxide at 37°C. BMSCs were harvested after they reached approximately 80% confluence. The PLGA/GelMA/IKVAV/USPIO composite scaffold was successively soaked in 75% alcohol (Dashti et al., 2016), sterile PBS and sterile DMEM/F12 with GlutaMAX for 1 h, respectively. Then, the BMSCs cells with a density of 1 × 10^5^ cells/ml were directly seeded onto the outside surface of sterilized PLGA/GelMA/IKVAV/USPIO composite tubular scaffold for routine cell culture.

### Characterization of GelMA

The infrared (IR) spectra of the Gel and GelMA was detected by Fourier transform infrared spectroscope (FTIR, TENSOR 27; Bruker, Germany). Rheological tests of GelMA hydrogel at different concentrations were performed using a strain-controlled rheometer (MCR 302, Anton Paar, Japan) equipped with a parallel-plate (25 mm) geometry. The storage modulus (G′) and loss modulus (G″) were both measured in this study. Oscillation time sweep was measured with 1 Hz frequency at 25°C for 600 s. Additionally, frequency sweep test was conducted from a function angular frequency of 0.1–10 Hz with 0.1% strain.

### Scanning Electron Microscope (SEM) Analysis

The micromorphology of the 3D structure scaffolds before and after incubating with GelMA hydrogel was observed by SEM (J SIRION-100; Eindhoven Netherlands) at 10 kV accelerating voltage. The samples were sputter coated with gold for 10 s before SEM observation.

### Mechanical Testing

The mechanical properties of PLGA/GelMA/IKVAV/USPIO scaffold (60 mm in length) was measured by a universal material testing machine (Instron 5543A, Massachusetts, United States) with a crosshead speed of 30 mm/min. Rectangular specimens of PLGA/GelMA/IKVAV/USPIO scaffold (20 mm in height) were measured at the compressive velocity of 1 mm/min. The compressive modulus was measured from 2 to 10% of linear curve fit from the compressive curve. At least three samples were recorded for final statistical evaluation.

### Swelling Testing

Swelling of the 3D print PLGA/GelMA/IKVAV/USPIO scaffold was investigated by the gravimetry method (Xue and Falcon, 2019). Briefly, lyophilized scaffold was weighed (*W*_d_) before being immersed in 2 ml PBS at 37°C until the equilibrium swelling was reached, after which the scaffold were removed. Excess surface water on the scaffolds was gently wiped with filter paper before the swollen scaffold was finally weighed (*W*_s_). The equilibrium swelling ratio was calculated using the following equation

$$\mathrm{Swelling}\,\mathrm{ratio}=\left(W_{\text{s}}-W_{\text{d}}\right)/W_{\text{d}}\times 100\%$$

### *In vitro* Biodegradation

The degradation studies of the fabricated 3D printing scaffolds (PLGA and PLGA/GelMA/IKVAV/USPIO) were conducted in PBS with or without lysozyme (Hu et al., 2019). Briefly, the tested scaffold samples (Wi) were immersed into 10 mL of PBS with or without lysozyme, followed by incubation at 37°C with a stirring rate of 60 rpm. At predetermined time points, the weights (Wc) of degraded samples were recorded after distilled water washing and freeze-drying.

The degradation rate was determined using the following equation:

Degradation=(Wi-Wc)/Wi×100%

where Wi is the weight of the scaffold at day 0, and Wc is the weight of the scaffold at each time point.

### MRI

First, the PLGA/GelMA/IKVAV/USPIO composite scaffold was placed in the centrifugal tube, and then all the composite tubular stents were performed T2 weighted imaging (T2WI) in clinical 3T whole body magnetic resonance scanner (SENSE-flex-M; Philips, Best, The Netherlands) using a small extremity coil. The parameters of T2WI were as follows: TR = 5000ms, TE = 5.8 ms, FOV = 8mm × 8mm, matrix size = 64 × 64, in-plane resolution = 125 mm × 125 mm, slice thickness = 0.8 mm.

### Effect of IKVAV on BMSCs

#### Effect of IKVAV on Adhesion Rate of BMSCs

The IKVAV were dissolved in PBS, at five concentrations (80, 100, 200, 400, and 800 μg/mL) to be used. Soak the round cover glass slides in 75% ethanol for 4 h, dry them in a 12-well plate, and coating in several different concentrations of IKVAV solutions. After incubated for 4 h in incubator and washed with PBS. 0.5 mM Laminin was used as positive control group, and the untreated group served as the negative control. BMSCs cells were inoculated into 12-well plate with 4 × 10^5^ per well. After 24 h, the cells were digested, counted, and statistical analysis was made. Cell adhesion rate was calculated according to the following formula:

Thecelladhesionrate(%)=(theadhesivecells)/(thetotalcell)×100%

BMSCs cells were inoculated into 96 plates at a density of 2 × 10^4^/ml for 24 h. Then, different concentrations of IKVAV solution (80, 100, 200, 400, and 800 μg/mL) were added, and three multiple holes were set up. At the period’s time point (1, 2, and 3 days), the OD value of cells was measured by CCK-8 kit to directly reflect the proliferation of cells. Cell viability was calculated according to the following formula:

$$\mathrm{Cell}\mathrm{viability}\left(\%\right)=\left({\text{OD}}_{\text{sample}}-{\text{OD}}_{\text{blank}}\right)/\left({\text{OD}}_{\text{control}}-{\text{OD}}_{\text{blank}}\right)\times 100\%$$

### Effect of USPIO of BMSCs

The GelMA hydrogels containing different concentrations of USPIO were prepared with a different mass/volume (g/L) ratio (USPIO: GelMA = 1:1, 2:1, 3:1, 4:1, 5:1, 6:1, 7:1, 8:1, 9:1, 10:1). Then, BMSC cells with the cell density of 1 × 10^4^ cells/mL were cultured and inoculated into 96-well plate. After the cells adhered to the wall, GelMA hydrogels with different concentrations of USPIO were added. After 24 h of culture, the medium was replaced with the DMEM solution containing 10% CCK-8, and the culture continued for 2–4 h. Under the condition of 450 nm wavelength, the absorbance of each hole was read by enzyme labeling instrument.

### Cell Proliferation Analysis

CCK-8 was used to assess the cell proliferation according to the instructions. BMSCs cultured with 10% fetal bovine serum and 1% penicillin/streptomycin at 37°C, 5% CO_2_ condition. Before culturing the cells, the PLGA/GelMA/IKVAV/USPIO scaffolds were soaked in 75% ethanol for 1 h, washed with PBS for 3 times, and then cultured overnight in a 24-well petri dish. The naked GelMA, PLGA scaffold and PLGA/GelMA/USPIO were used as control. On days 1, 4, 7, and 10 of culture, samples were washed 3 times with PBS. Then, the DMEM medium containing 10% volume of CCK-8 were added to each well, and incubated for another 3 h. Finally, the OD of each solution were evaluated by microplate reader among at wavelength of 450 nm.

Live and dead cells were further visualized by LIVE/DEAD cell kit. The activity of intracellular esterase and the integrity of plasma membrane were measured by Calcein AM and Ethidium homodimer-1, respectively. They were mixed with phosphate buffer saline (DPBS) of Dulbecco and added to media-free cells/scaffolds, and the cells on scaffolds were observed by an inverted fluorescence microscope.

### Cell Morphology Analysis

The morphology of cells was observed by cytoskeleton staining, including phalloidin staining and DAPI staining. Briefly, BMSCs cells were inoculated into PLGA/GelMA/IKVAV/USPIO scaffolds with an initial density of 4 × 10^4^/cm^2^ for 3 days. Then, the scaffold was taken out of the wells, fixed with 4% paraformaldehyde for 30 min, and washed with PBS. Finally, cells on the scaffold were counterstained by the F-actin with Phalloidin and nuclei with dye diamidino-2-phenylindole (DAPI). Finally, cells on the scaffold were observed by a confocal laser microscope (Leica, Wetzlar, Germany).

### Animal Experiments

The animal experiments were performed with the permission of the Institutional Animal Care and Use Committee (IACUC) of Central South University. The male inbred Wuzhishan miniature pigs at 8 months of age weighing 30 ± 2.5 kg were Haikou Peoples Hospital (Hainan, China). All animals were maintained under 12-h light/dark conditions and fed a standard commercially available pig feed. Animals were divided into two groups (Normal and PLGA/GelMA/IKVAV/USPIO composite tubular scaffold treated) of six animals each. Before surgery, animals were fasted overnight for 12 h with access to water only. The pigs were fasted overnight for 12 h prior to surgery, and were provided with oral antibiotics (300 mg of cephalexin) following surgery. All pigs were anesthetized, placed in ventral recumbency, and laparotomized via a midline incision in the upper abdomen to expose the extrahepatic bile duct (EHBD) (Figure 9A). The lower EHBD was transected and the artificial PLGA/GelMA/IKVAV/USPIO composite bile duct scaffold was anastomosed end-to-end to the proximal and distal ends of the EHBD with interrupted 4.0 Ethilon sutures (Figure 9B). We further confirmed adequate bile drainage through the anastomosed bile duct scaffold during the surgery, the abdominal cavity was irrigated with warm saline and closed following surgery. For histological analysis, fresh tissues obtained from the pig bile duct were fixed with 10% formalin followed by paraffin embedding. Samples were sectioned at 4 μm and stained with hematoxylin and eosin (H&E). For immunohistochemistry, sections were blocked with 5% bovine serum albumin (BSA) in PBS for 30 min and incubated overnight with the primary antibody CK19 (BA4154, 1: 400, Boster) in PBS containing 1% BSA at 4°C.

### Statistical Analysis

All of the experiments were carried out at least in triplicate. All data were presented as mean result ± standard deviation (SD). One-way analysis of variance (ANOVA) evaluated the differences between groups. *P* < 0.05 considered statistically significant, with ^∗^ = *p* < 0.05; ^∗∗^ = *p* < 0.01; ^∗∗∗^ = *p* < 0.001.

## Results and Discussion

### Characterization of GelMA

**FIGURE 1:**
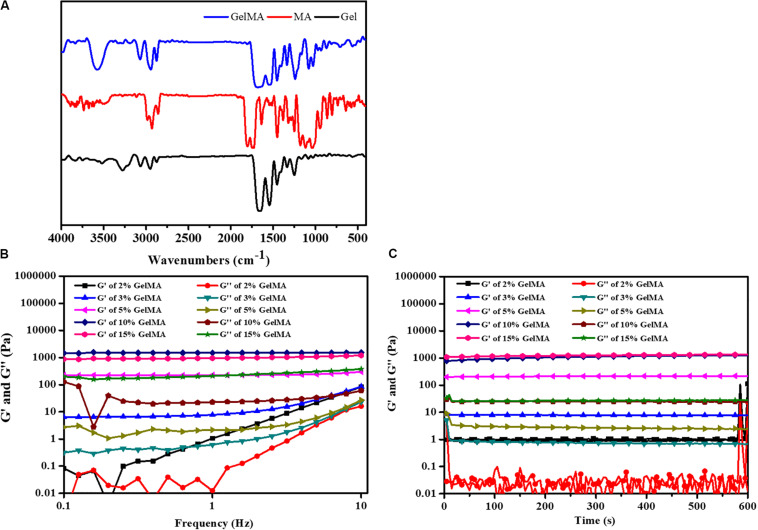
**(A)** FTIR spectrum of GelMA. **(B)** The gel viscosity with frequency ranging from 0.1 to 10 Hz. **(C)** Rheological properties.

To form the hydrogel for bile duct restoration, we synthesized the photocrosslinkable GelMA polymer by reacting methacrylic anhydride with gelatin according to methods previously described (Sun et al., 2018; Aldana et al., 2019b). The FTIR spectra of gelatin, MA and GelMA were shown in Figure 1A. The main bands of gelatin included a strong broad overlapping peak of 3252 cm^–1^, attributed to the stretching of O–H (Topkaya, 2015). In addition, the characteristic absorption bands of GelMA were observed 1697 cm^–1^ (amide I, the stretching of C=O bond), 1542 cm^–1^ (amide II, bending of N-H bond) and 1458 cm^–1^ (amide III, plane vibration of C–N and N–H), respectively (Aldana et al., 2019a). Meanwhile, N–H stretching (amide A) was observed at 3284 cm^–1^, demonstrating that MA was successfully grafted onto the gelatin chains.

To demonstrate the feasibility of the formed bioink for 3D printing, the rheological behaviors were carried out. The gelation behavior of different concentrations of GelMA was monitored by rheological analysis. As shown in Figure 1B, the storage modulus (G′) also surpassed the loss modulus (G″) with the frequency from 0.1 to 10 Hz, which indicated that the photo-crosslinked GelMA hydrogel had enhanced resistance to deformation. As shown in Figure 1C, the time-sweep oscillation experiment shows the storage modulus G’ of hydrogels was increased with an increase in the concentration of the GelMA. There was no significant difference between the G′ of 15% GelMA and the G′ of 10% GelMA. However, GelMA hydrogels at concentrations of 2 and 3% (w/v) with low G′ (∼100 Pa) was insufficient to enhance the mechanical strength of the PLGA scaffold. Noticeably, GelMA hydrogels with a concentration of 10% and 15% (w/v) have a high G′ (∼500 Pa), which is similar to a previous study (Athirasala et al., 2017).

### Characterization of the Scaffold

**FIGURE 2:**
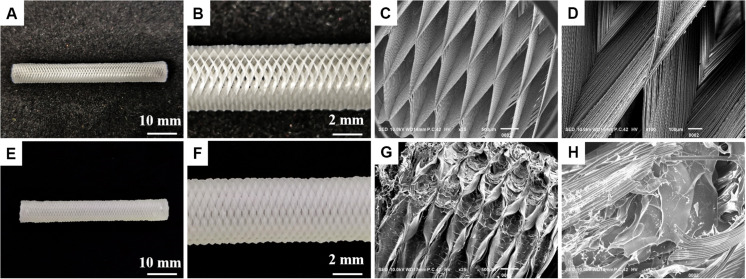
Digital image of PLGA **(A,B)** and **(E,F)** PLGA/GelMA/IKVAV/USPIO tubular scaffold. Low- and high-magnification SEM image of PLGA **(C,D)** and PLGA/GelMA/IKVAV/USPIO **(G,H)**.

An ideal hydrogel-based scaffold for usage in tissue engineering and regenerative medicine should have the following characteristics, such as sufficient mechanical properties, appropriate swelling ratio and biodegradation ratio *in vivo*, and good biocompatibility (Kim et al., 2016). Therefore, we systematically studied the effect of adding GelMA on the performance of these scaffolds. Here, a conduit with the fiber diameter of ∼50 μm was created using microneedles. After curing, the microneedles and the rolling tube mold were removed. The morphology of the 3D printing conduit was characterized using optical imaging and scanning electron microscopy (SEM). As shown in Figures 2A–D, the PLGA tubular scaffold has a uniform rhombohedral porous structure, and the wire diameter was uniform, about 5–7 μm, which was beneficial to the adhesion and growth of cells. The GelMA/IKVAV/USPIO gel uniformly fills the pores of the PLGA tubular scaffold. As shown in Figures 2E–H, the surface of PLGA/GelMA/IKVAV/USPIO composite tubular scaffold has a microporous structure, which was beneficial to cell adhesion and growth (Shkarina et al., 2018).

### Mechanical Properties of the Scaffolds

**FIGURE 3:**
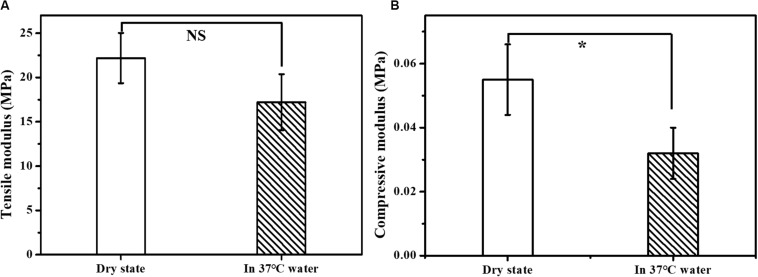
Mechanical properties of the composite tubular scaffold. **(A)** Tensile modulus and **(B)** compression modulus of PLGA/GelMA/IKVAV/USPIO composite tubular scaffold in dry state (air environment) and wet state (37°C water bath).

An ideal scaffold should equip with good mechanical properties to keep its integrity during use. The mechanical properties of the prepared scaffolds were evaluated by the tensile modulus and compressive modulus. As shown in Figure 3, the tensile modulus of the scaffold was measured to be in the range of 17.19 – 29.05 MPa and the compressive modulus in the range of 0.042 – 0.066 MPa. The mechanical properties were also tested in 37°C water bath to simulate the bile flow environment *in vivo*. The measured tensile modulus of the multi-layer 3D-fabrication of PLGA/GelMA/IKVAV/USPIO scaffold was in the range of 14.05 – 20.37 MPa and the compressive modulus in the range of 0.024 – 0.04 MPa, which was suitable for bile duct implantation. Previous research also reported that the mechanical strength of the pure PLGA scaffold was similar to a real bile duct (Zong et al., 2017). The mechanical test indicated that the PLGA/GelMA/IKVAV/USPIO 3D conduit has both flexibility and toughness, which provide a supporting structure and allow duct regeneration. Furthermore, the designed bilayered scaffold has porous structure and improved mechanical stability. Additionally, the bilayered scaffold can be used to design artificial arteries and intestines (Zong et al., 2017).

### Swelling Study

**FIGURE 4:**
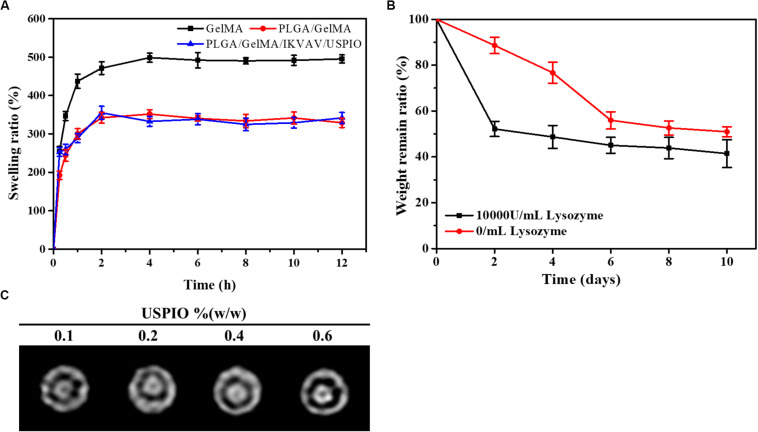
Physical properties of the composite tubular scaffold. **(A)** Swelling ratio of GelMA, PLGA/GelMA, and PLGA/GelMA/IKVAV/USPIO. **(B)** Weight loss rate of the PLGA/GelMA/IKVAV/USPIO scaffolds in PBS with or without 10000 U/mL lysozyme. **(C)** *In vitro* MRI imaging of PLGA/GelMA/IKVAV/USPIO composite tubular scaffold.

As shown in Figure 4A, the time-dependent swelling ratio was investigated by using water uptake experiment. The Swelling ratio of the GelMA were around 498.7 ± 11.8%, which was related to the high hydrophilicity of GelMA and porous structure of scaffold. The swelling ratio of PLGA/GelMA and PLGA/GelMA/IKVAV/USPIO scaffold was 351.7 ± 11.5%, 355.2 ± 17.0%, respectively. The relatively low swelling ratio may be related to the presence of PLGA and USPIO which has hydrophobic groups could decrease water adsorption. Meanwhile, PLGA and USPIO layer changed the internal structure and decrease the proportion of micropore, resulting in low swelling ratio. This low swelling ratio will not cause collateral damage to normal tissues.

### Stability of Bilayered Bile Duct Scaffold

Recently, most of the bile duct stents used in clinic were non-degradable scaffolds, but as a long-term foreign material, non-degradable scaffolds were easy to cause inflammation, restenosis and other phenomena, resulting in secondary implantation difficulties. In addition, patients have to endure the pain of scaffold removal. Therefore, an increasing amount of scaffolds for bile duct repair were reported (Miyazawa et al., 2005). However, few studies on bilayered bile duct scaffolds have been reported at this time. Here, a kind of PLGA/GelMA bilayered bile duct scaffold was designed and fabricated. The PLGA inner layer exhibited a smooth and compact surface, which will promote the bile acid flow and avoid the bile leakage or cholestasis after implantation. The GelMA outer layer was able to effectively support cells adhesion and growth, load bioactive substances and accelerate the formation of new tissue. It would require as long as 2 years to fully degrade the entire PLGA scaffold *in vivo* (Sun et al., 2006). However, prior study indicated that GelMA hydrogel were completely degraded in PBS after 2 weeks (Yu et al., 2020). As shown in Figure 4B, weight retention rate of the PLGA/GelMA/USPIO/IKVAV scaffold either in PBS alone or PBS contained lysozyme decreased gradually with the incubation time increased. Despite the fact that enzyme do not exist in PBS, the hydrogel discs might be degraded by hydrolysis. The degradation of hydrogel were due to a combination of enzymolysis and hydrolysis. Moreover, the prepared scaffold shown a faster degradation behavior in PBS with lysozyme. The PLGA/GelMA/IKVAV/USPIO scaffold degraded ∼40% after 10 days, the fast degradation rate was associated with lysozyme. Prior study reported that lysozyme could degrade natural degradation sites for collagenases and matrix metalloproteinases (MMPs) in GelMA (Yu et al., 2020).

### MRI

The composite scaffold was visualized by incorporating USPIO as contrast agent. As shown in Figure 4C, the different concentration of USPIO in PLGA/GelMA/IKVAV/USPIO composite tubular scaffold showed different degree of T2-weighted MRI imaging. MRI displayed a uniform signal at the boundary regions, which could intuitively see the original shape and status of the composite scaffold. Moreover, it can be seen that with the concentration of USPIO increases, the more obvious the imaging effect becomes. Hence the position changes of the fluorescence of USPIO can be used to monitor degradation of scaffold in real time (Lei et al., 2017). Previous research also reported that T2-weighted MRI imaging was stable and reasonable, USPIO-labeled scaffcan be monitored in real time (Chen et al., 2018).

### Effect of IKVAV of BMSCs

#### Effect of IKVAV on Adhesion Rate of BMSCs

**FIGURE 5:**
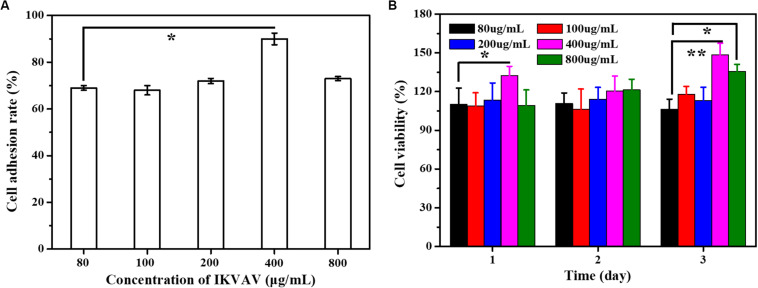
**(A)** The adhesion rate of the cells with different IKVAN concentrations. **(B)** The cell viability with different IKVAV concentrations.

The surface modification of biomedical scaffolds can enhance the adhesion of cells to materials and the biocompatibility of tissues and materials. One way to enhance cell adhesion was to use bioactive peptides such as collagen, fibronectin and laminin, which exist in extracellular matrix, which promote cell adhesion. For example, P15, a polypeptide from collagen, can promote cell adhesion and modulate a variety of genes (Carinci et al., 2004; Lucidarme et al., 2004; Funfak et al., 2019). The peptide has been integrated into a dental implant material and achieved good results. Other peptides that also promote cell adhesion include RGD, YIGSR and IKVAV, from laminin and peptides from laminin, which can directly promote cell adhesion (Kang et al., 2019). The effect of IKVAV on adhesion rate of BMSCs was shown as Figure 5A. Through the statistics of cell count at several time points, it can be seen intuitively that although the treatment of IKVAV was not as strong as that of Laminin, it was significantly higher than that of the negative control group, indicating that it still has the effect of promoting cell adhesion to a certain extent. In addition, it can also be seen that the cell adhesion rate at 400 μg/mL was higher than that of other concentrations, and the cell survival rate at this concentration was higher, second only to the positive group.

#### Effect of IKVAV on Proliferation of BMSCs

The effects of IKVAV on the proliferation of BMSCs were measured by CCK-8 and the results as shown in Figure 5B. At the same time point, the cell survival rate increased with the increase of polypeptide concentration, but when the concentration was higher than 400 μg/mL. On the contrary, the survival rate of cells decreased. In addition, it can also be seen that at the same concentration, the survival rate increases with the increase of time.

### Effect of USPIO on BMSCs

**FIGURE 6:**
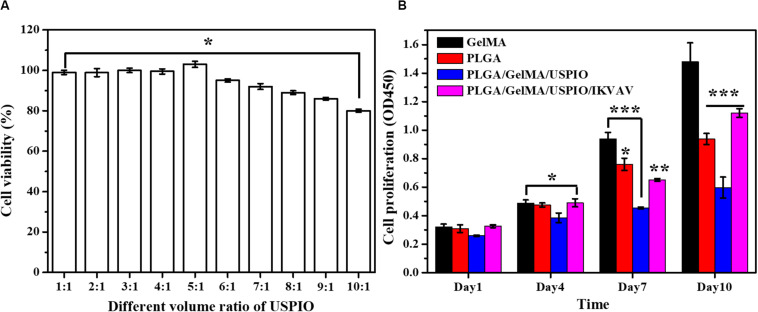
*In vitro* biocompatibility evaluation of the composite tubular scaffold. **(A)** The cell viability of GelMA/USPIO with different USPIO concentrations. **(B)** Proliferation of BMSCs seeded on various composite scaffolds at day 1, 4, 7, and 10.

As shown in Figure 6A, when the volume ratio of GelMA: USPIO was 5: 1, the cells have the highest survival rate, and when the volume ratio was less than 5: 1, the cells showed toxic effects. The higher concentration of USPIO, the better the MRI imaging effect. Therefore, under the condition of guaranteeing cell survival, GelMA hydrogel with a large concentration of USPIO was selected, that is, the preferred GelMA and USPIO volume ratio was 5:1.

### Cell Proliferation Analysis

The biocompatibility of the developed scaffold was assessed by measuring the proliferative state of BMSCs on the surface of scaffold. The cell viability and proliferation of different scaffold for 1, 4, 7, and 10 days as quantitatively examined by CCK-8. As shown in Figure 6B, the cell proliferation of pure GelMA group increased significantly from day 1 to day 10. Noticeably, PLGA and PLGA/GelMA/USPIO group showed a relatively lower cell proliferation rate than the GelMA group at day 7 and day 10. It was mainly due to the interaction of PLGA and USPIO with the cells. However, PLGA/GelMA/USPIO/IKVAV showed a higher proliferation than the PLGA and PLGA/GelMA/USPIO group at day 10, which attributed to the positive effect of GelMA and IKVAV modified PLGA in cell proliferation. Based on these results, it was concluded that the PLGA/GelMA/IKVAV/USPIO composite tubular scaffold have excellent biocompatibility to be favorable for BMSCs growth and proliferation.

**FIGURE 7:**
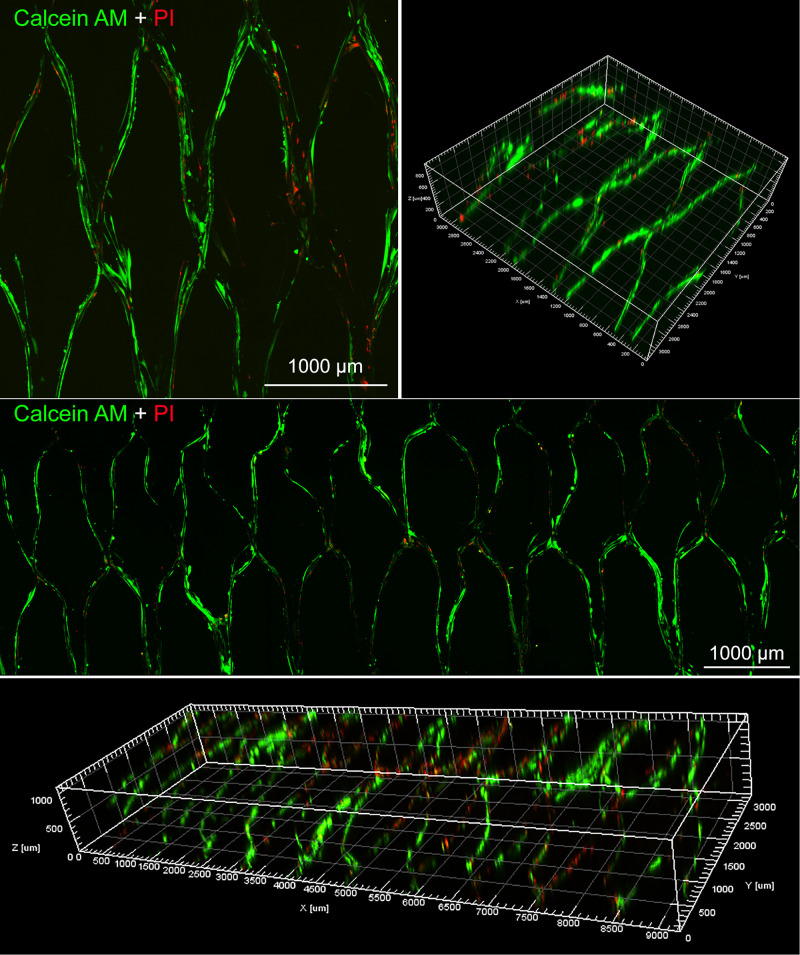
Live/dead staining images of BMSCs seeded on PLGA/GelMA/IKVAV/USPIO composite scaffold at day 10.

Moreover, the proliferation of BMSCs on the surface of various scaffolds were further quantitatively visualized by Live/Dead staining at day 10. As shown in Figure 7, most of the cells were alive in PLGA/GelMA/IKVAV/USPIO composite tubular scaffold. Moreover, PLGA/GelMA/IKVAV/USPIO group exhibited high cell density and survival rate of over 90%, which was consistent with the results of cell proliferation. Hence, we designed 3D printed scaffolds to improve both cellular distribution and proliferation efficiencies of cells within the scaffold. Therefore, GelMA based 3D printed scaffolds with a satisfied cell density have a great potential for tissue engineering.

### Cell Morphology Analysis

**FIGURE 8:**
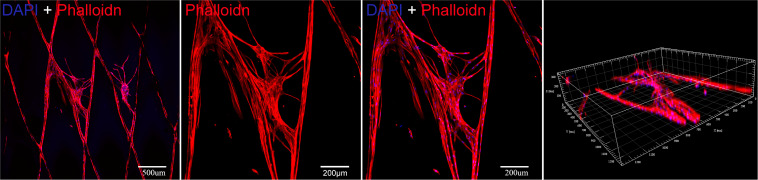
Cytoskeleton fluorescence staining image of BMSCs seeded on a PLGA/GelMA/IKVAV/USPIO composite scaffold at day 13.

As shown in Figure 8, BMSCs presents a large cytoskeleton (red stained by Phalloidin reagent) and a small dotted nucleus (blue stained by DAPI reagent). After 13 days in culture, the cells can be well adhered and spread in PLGA/GelMA/IKVAV/USPIO composite tubular scaffold. In addition, the cells can be seen sticking out on the material and adhering to the surface of the material. Meanwhile, a large number of cells grown into the internal fiber of composite tubular scaffold. The results showed that PLGA/GelMA/IKVAV/USPIO composite tubular scaffold can provide larger surface areas for cell adhesion.

**FIGURE 9:**
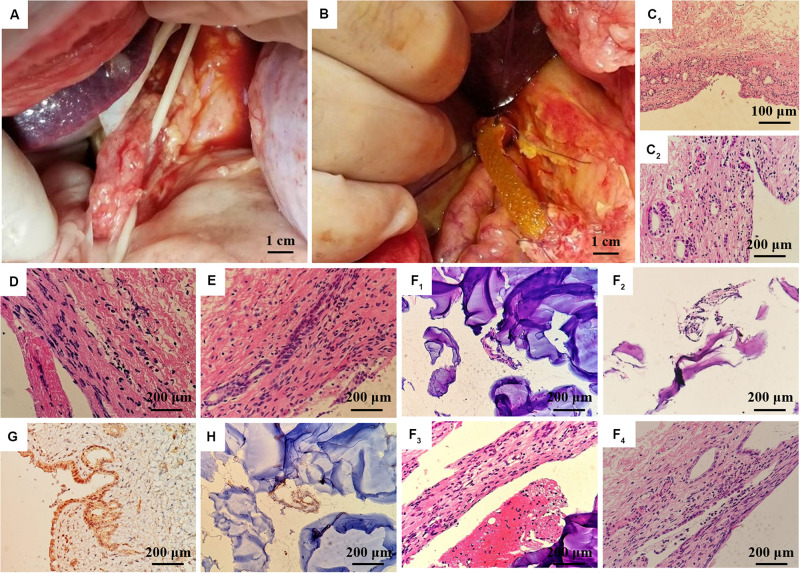
*In vivo* regeneration of pig bile duct. **(A)** Free bile duct of inbred wuzhishan miniature pig. **(B)** PLGA/GelMA/IKVAV/USPIO composite stent implantation procedure. **(C)** HE staining of common bile duct of normal inbred (**C1**: 200×, **C2**: 400×). HE staining of common bile duct in the experimental group **(D)** and the sham operation group **(E)** at 14 days after surgery. **(F)** HE staining of common bile duct and PLGA/GelMA/IKVAV/USPIO composite scaffolds at 14 days after surgery. **(G)** Normal bile duct epithelial cytoplasm and **(H)** composite scaffolds immunohistochemical staining of bile duct CK19 at 14 days after surgery.

### *In vivo* Pig Bile Duct Defect Model

Histological analysis was performed on the surgically treated bile duct with hematoxylin and eosin (H&E). As shown in Figure 9C, the normal common bile duct showed that the histological structure was similar to that of human tissue. The bile duct wall was divided into three layers (mucous layer, muscular layer and adventitia layer) from inside to outside, and the boundary of each layer was not very clear. The surface was lined with a single layer of columnar mucosal epithelium and the basement membrane was not obvious. The lamina propria is dense fibrous connective tissue, which contains a lot of collagen and elastic fibers, and small blood vessels, in which a few single tubular submucosal glands can be seen. The distribution of smooth muscle in each segment of bile duct was different. The smooth muscle in the upper part of the bile duct was scattered or absent, while the smooth muscle bundles in the lower part of the bile duct were continuous or intermittent, and the middle part was between them, and the muscle bundles were mainly fibrous connective tissue. The adventitia is loose connective tissue including lymphatic vessels, nerve fibers and ganglion cells.

At 14 days after operation, HE staining PLGA/GelMA/IKVAV/USPIO composite tubular scaffold group showed that the morphology and structure of bile duct was normal, the small vessels in the lamina propria of bile duct were dilated, neutrophils and lymphocytes were infiltrated, and inflammatory cellulose-like exudate was seen on the surface of the stent (Figures 9E,F). A mild meutrophil and lymphocyte infiltration, epithelioid cell and multinucleated giant cell infiltration were seen on the surface of the scaffold. HE staining in control group showed normal morphology and structure of bile duct and infiltration of neutrophils and lymphocytes in the control group (Figure 9D).

At 14 days after operation, the immunohistochemical staining showed that the expression of CK19 in bile duct epithelial cells was positive. The expression of CK19 in bile duct epithelial cells was positive in normal group (Figure 9G). CK19 positive cells could be seen in the anastomotic site and inside the PLGA/GelMA/IKVAV/USPIO composite tubular scaffold, indicating the formation of bile duct-like epithelial cells (Figure 9G). A small amount of CK19 positive tissue with tubular arrangement could be seen on the composite scaffold, indicating the regeneration of tubular tissue (bile duct).

## Conclusion

This study presents a novel 3D printing PLGA/GelMA/IKVAV/USPIO duct conduits through the layer by layer casting (LBLC) method. Different from traditional electrospinning fabrication of duct conduit, 3D printing technique avoided many disadvantages, such as the inconsistencies of temperature control, poor mechanical strength and gaps between nanofibers. Moreover, 3D printed scaffold can be used for real-time non-invasive detection of bile duct repair and scaffold degradation through MRI. The IKVAV were uniformly distributed on the duct conduit by the LBLC method to assure the good biocompatibility for duct regeneration. *In vivo* pig bile duct defect model, the tailored shape of the artificial duct PLGA/GelMA/IKVAV/USPIO duct conduits proved that its feasible and practicability in surgery. HE and immunohistochemistry showed that a mild meutrophil and lymphocyte infiltration, epithelioid cell and multinucleated giant cell infiltration with no evidence of bile leakage into the surrounding tissue, and the presence of CK19 expression. The finding of this study demonstrates that the prepared bilayered PLGA/GelMA/IKVAV/USPIO conduits can be used as a promising candidate for bile duct restoration and detection. Moreover, the present strategy provides a new method in the field of bilayered conduits for cell culture and tissue engineering.

## Data Availability Statement

All datasets generated for this study are included in the article/supplementary material.

## Ethics Statement

The animal study was reviewed and approved by the Institutional Animal Care and Use Committee (IACUC) of Central South University.

## Author Contributions

YX carried out development of hydrogels, rheology, and 3D-fabrication of PLGA, and performed the mechanical testing. YX and WW carried out evaluation of scaffolds. YX and YY carried out the cell studies. All authors contributed to the article and approved the submitted version.

## Conflict of Interest

The authors declare that the research was conducted in the absence of any commercial or financial relationships that could be construed as a potential conflict of interest.
